# Aqueous Extract from *Cinnamomum zeylanicum* (Lauraceae) Stem Bark Ameliorates Gentamicin-Induced Nephrotoxicity in Rats by Modulating Oxidative Stress and Inflammatory Markers

**DOI:** 10.1155/2021/5543889

**Published:** 2021-07-19

**Authors:** Albert Donatien Atsamo, Auscar Lontsie Songmene, Mireille Flaure Metchi Donfack, Omer Bébé Ngouateu, Télesphore Benoît Nguelefack, Théophile Dimo

**Affiliations:** ^1^Department of Animal Biology and Physiology, Faculty of Science, University of Yaoundé I, P.O. Box 812, Yaoundé, Cameroon; ^2^Department of Animal Biology, Faculty of Science, University of Dschang, P.O. Box 67, Dschang, Cameroon

## Abstract

Nephropathies and especially nephrotoxicity have become one of the serious causes of life-threatening conditions because of intensive exposure to xenobiotic whether by environmental pollution or by drug abuse. The present study was undertaken to assess the protective effects of *Cinnamomum zeylanicum* stem bark aqueous extract (AECZ) on gentamicin-induced nephrotoxicity. AECZ was prepared by maceration in water and tested orally at the doses of 200 and 400 mg/kg/day to prevent gentamicin-induced nephropathies in male Wistar rats. Gentamicin (100 mg/kg/day) was administered for 14 consecutive days by intraperitoneal route, concomitantly with AECZ or silymarin (50 mg/kg/day) used as reference drug. Animal body weight was monitored during the treatment. After the last treatment on the 14th day, animals were sacrificed. Blood was collected for the evaluation of hematological and renal function biomarkers. The homogenate of one kidney was used to assess oxidative stress markers and proinflammatory cytokines, while the other one was fixed in formaldehyde for histopathological studies. Gentamicin decreased body weight, serum total proteins, and calcium level but increased kidneys' relative weight, serum creatinine, urea, and uric acid. Moreover, the levels of reduced glutathione, catalase, and superoxide dismutase activities were decreased, while an increase in malondialdehyde, proinflammatory cytokines (TNF-*α*, IL-1*β*, and IL-6), and nitrites was observed in the negative control group as compared to normal control. Histological analysis of the kidney revealed the presence of tubular necrosis, glomerular degeneration, and macrophage infiltration in the gentamicin-treated group. All these impairment parameters were prevented by AECZ and silymarin treatments. AECZ has a protective effect against gentamicin-induced nephrotoxicity. The antioxidant and anti-inflammatory potentials of this extract may highly contribute to its nephroprotective activity.

## 1. Introduction

Drug-induced nephrotoxicity is one of the common problems in clinical medicine. Nephrotoxic medications can lead to damage to the kidney via various mechanisms, including structural and functional alterations. When evaluating the primary causes in renal injury, the incidence of drug-induced toxicity has accounted for 20% of all-cause incidents [1, 2]. Many drugs, including gentamicin (GM), are nephrotoxic. Gentamicin is an aminoglycoside antibiotic agent that is widely used in clinical practice for the treatment of Gram-negative infection [3]. However, 10 to 30% of patients subjected to this drug have high risk of developing renal dysfunction mainly after long-term treatment [1, 2]. Renal injury induced by gentamicin is related to its preferential accumulation in the renal proximal convoluted tubules, which leads to loss of brush border integrity [4]. Mechanisms of GM-induced nephrotoxicity comprise free radicals generation, increase lipids peroxidation, and decrease activity of endogenous antioxidant, renal inflammation characterized by macrophage infiltration, and subsequent release of proinflammatory cytokines associated with the activation of the stress-induced NF-*κ*B, acute tubular necrosis, and glomerular congestion, resulting in diminished glomerular filtration rate and renal dysfunction [5–7]. Several approaches have been tried to reduce gentamicin nephrotoxicity by using various natural antioxidants, such as the use of *Moringa oleifera* seeds, *Rosmarinus officinalis*, and *Thymus vulgaris* [8, 9]. This is due to their effectiveness, minimal side effects in clinical use, and relatively low costs. Herbs have many phytochemicals which possess antioxidant activity and can be promising agents against gentamicin toxicity.

*Cinnamomum zeylanicum* (Lauraceae), also known as true cinnamon, which originates from Sri Lanka and south India, is a tree that is about 10 meters high and can withstand hard climatic conditions. It is found predominantly in tropical and subtropical regions, where the rainfall and the temperature range from 1500 mm to 2500 mm and from 27°C to 30°C, respectively [10]. Their leaves and stem barks are important and popular spices used worldwide not only for cooking but also in traditional and modern medicines [11].

Many communities in Cameroon use the stem bark of this plant traditionally for the management of various ailments such as muscular pain, rheumatism, gastrointestinal disorders, and typhoid fever [12]. It is also used as an aphrodisiac and an antihypertensive medicine [13]. Pharmacological investigations carried out on *C. zeylanicum* have demonstrated its therapeutic potentials including antihypertensive and vasorelaxant activities [13], antinociceptive and anti-inflammatory effects [14], antidiabetic activity [15], immunomodulatory activity [16], and antiartherosclerotic potential against glucocorticoid-induced atherosclerosis in rat [17]. The methanol extract of *Cinnamomum zeylanicum* stem bark also demonstrated acute and chronic antihypertensive properties in NO-deficient hypertensive rats [18]. Recently, research has found that the aqueous extract of *Cinnamomum zeylanicum* stem barks can attenuate diclofenac sodium and oxytetracycline mediated hepatorenal toxicity by modulating oxidative stress, cell apoptosis, and inflammation in male albino rats [19]. Also, cinnamon oil protects kidney against acetaminophen-induced nephrotoxicity by ameliorating oxidative stress, apoptosis, and inflammation in rats [20].

The stem barks of *Cinnamomum zeylanicum* contain 45%∼65% cinnamaldehyde, 12%∼18% eugenol, and small amounts of 2′-hydroxycinnamaldehyde and 2′-benzoyl-oxycinnamaldehyde [21, 22].

Cinnamaldehyde, a phenolic compound primarily identified as the main phytochemical found in cinnamon bark, is responsible for most of the biological effects of *Cinnamomum zeylanicum* [23]. Various studies have suggested that antifungal, anticancer, antimutagenic, anti-inflammatory, neuroprotective, and antioxidant effects of *Cinnamomum zeylanicum* are mainly due to the presence of cinnamaldehyde and eugenol [24, 25]. It is well established that extracts and oils obtained from cinnamon have strong free radical scavenging or antioxidant activity related to the presence of flavonoids and polyphenolic compounds [12, 26].

Although the nephroprotective activity of the ethanolic extract of *Cinnamomum zeylanicum* stem barks has been demonstrated [27], no information is presently available as regards that of the aqueous extract, which is the most common form of preparation used by the population. Besides, the mechanisms by which *C. zeylanicum* protects against kidney damage are not known. The present study was undertaken to determine the preventive effect of AECZ extract in gentamicin-induced nephrotoxicity by investigating the impact of treatment on some renal biomarkers, oxidative stress parameters, proinflammatory cytokines, and histopathological features.

## 2. Materials and Methods

### 2.1. Plant Material and Preparation of the Extract

*Cinnamomum zeylanicum* parts were harvested in Njombe (Littoral Region of Cameroon) in August 2018 and used for the authentication of the plant in Yaoundé National Herbarium by comparison with the voucher specimen SRFC/22309. The stem barks were air-dried at room temperature and ground into a fine powder. 800 g of this powder was macerated into 7 L of distilled water for 48 hours. The mixture was then filtered with Whatman filter paper no. 3. The filtrate was lyophilized, and 30.78 g of the aqueous extract was obtained from this preparation (yield of 3.85%).

### 2.2. Animal Material

Thirty-six male, 8-9-week-old Wistar rats weighing between 135 and 145 g at the beginning of the experiment were obtained from the animal house of the Department of Animal Biology and Physiology, Faculty of Science, University of Yaoundé 1, Cameroon. They were maintained under standard laboratory conditions with natural light cycle and had free access to normal laboratory rat chow and tap water. The study was conducted in accordance with the guidelines of the Cameroon National Ethical Committee of the use of laboratory animals for scientific research (ref. NFWA-IRD 0001954).

### 2.3. Animals Grouping and Experimental Design

The thirty-six rats were randomized and assigned to six groups composed of six animals each and treated daily for 14 days as follows: a vehicle control group which received distilled water (10 mL/kg, *p.o.*) and saline solution 0.9% (1 mL/kg, *i.p.*), a negative control group which received distilled water (10 mL/kg, *p.o*.) and gentamicin (100 mg/kg, *i.p.*), a positive control group which received silymarin (50 mg/kg, *p.o*.) and gentamicin (100 mg/kg, *i.p.*), the two test groups which received the aqueous extract of *Cinnamomum zeylanicum* (AECZ) at the doses of 200 mg/kg and 400 mg/kg (*p.o*.) and gentamicin (100 mg/kg, i.p.), and the extract control group which was treated with AECZ (400 mg/kg/, p.o.) and saline solution 0.9% (1 mL/kg, *i.p*.). The doses of the extract were chosen from literature based on their protective effect against acetaminophen-induced cellular damage and apoptosis in renal tissue [28]. The oral administration of the plant extract or silymarin was performed one hour before the intraperitoneal injection of gentamicin. Body weight was recorded at the beginning and at the end of the experimental period.

At the end of the treatment on day 15, animals were fasted overnight but had free access to water. They were then anesthetized with urethane (1.5 g/kg, *i.p.*) and blood from the carotid arteries was collected using capillary tubes under an anticoagulant (Ethylenediaminetetraacetate) or not, for hematological and biochemical studies, respectively. After blood collection, the rats were sacrificed by decapitation. The two kidneys were collected and dry-blotted and the relative organ weight was calculated.

### 2.4. Measurement of Hematological Parameters

Hematological analysis was performed with blood collected under EDTA using a hematimeter (Mindray BC-3000). The parameters considered were the red blood cells (RBC) count, the white blood cells (WBC) count, the hemoglobin (Hb) concentration, the hematocrit (HCT), the mean corpuscular hemoglobin (MCH), the mean corpuscular volume (MCV), the mean corpuscular hemoglobin concentration (MCHC), the platelet count, the lymphocyte, the monocyte, and the granulocyte counts.

### 2.5. Biochemical and Histological Analysis

Serum was separated from the clotted blood sample by centrifugation (1510×g, 15 min, 4°C) and the collected sample was stored at −20°C for analysis. Glucose (CN : BXC0101A), albumin (CN : BXC0221A), aspartate aminotransferase (CN : BXC0128A), urea (CN : BXC0126A), creatinine (CN : BXC0117A), uric acid (CN : BXC0603B), and total calcium (CN : BXC0292A) were assayed in serum samples, using corresponding commercial diagnostic kits purchased from Fortress, UK. Total protein was evaluated using the method of Bradford (1976). Serum Na^+^, K^+^, and Cl^−^ were determined using automatic equipment (Biolyte 2000 - Na^+^, K^+^, Cl^−^/Li^+^, Diocare Corporation, Thailand).

One of the collected kidneys was homogenized at 20% (w/v) in Tris-HCl 50 mM buffer (pH 7.4). The obtained homogenates were centrifuged at 1510×g for 15 minutes at 4°C. The supernatant collected was stored at −20°C for further biochemical assays. The total protein was determined by the method of Bradford [29]. The superoxide dismutase (SOD) activity was assessed according to Misra and Fridovich. Reduced glutathione (GSH) and catalase were assayed following the method described by Ellman [30] and Sinha [31], respectively. Nitrites' content was estimated as described by Slack [32], while malondialdehyde (MDA) was determined by the method described by [33].

The levels of the inflammatory cytokines, interleukin-1*β* (IL-1*β*), IL-6, and tumor necrosis factor-*α* (TNF-*α*), in kidney homogenate were measured using a Sandwich enzyme-linked immunoassay kits specific to each cytokine (R&D Systems, USA). The measurements were performed at 450 nm using a microplate-reading spectrophotometer (Thermo Scientific Multiskan FC). The levels of TNF-*α*, IL-1*β*, and IL-6 were expressed as pg/mg of protein [34].

For histological analysis, the second kidney was fixed in 10% formalin for 7 days and further paraffin-embedded. Sections of 4–5 *μ*m were made and further stained with hematoxylin and eosin (H&E). The sections were examined with light microscope (Zeiss Axioscope) and digital camera, and MiniSee software was used for images capture. As an example of kidney damage, tubular clarifications were quantified using ImageJ software, version 1.48 for Windows. The percentage of tubular clarifications was calculated as follows:

(1) $$\begin{array}{c} \%\text{ tubular clarification}=\left(\frac{\text{tubular clarification count}}{\text{total tubular count}}\right)\times 100. \end{array}$$

### 2.6. Statistical Analysis

Data were expressed as mean ± standard error of mean. Statistical analysis was performed using one-way analysis of variance (ANOVA) followed by the Tukey post hoc test. *p* < 0.05 was considered statistically significant. All analyses were performed using GraphPad Prism software, version 8.01.

## 3. Results

### 3.1. Effect of *C. zeylanicum* on the Body Weight and Relative Kidney Weight

Gentamicin significantly (*p* < 0.001) decreased the rat weight gain by 45.93% and increased the relative kidney weight by 48.34% as compared to normal control group. AECZ at the doses of 200 and 400 mg/kg significantly (*p* < 0.001) prevented the decrease of weight gain and the increase of relative kidney weight by 14.13% and 17.39%, respectively, compared to gentamicin group. AECZ administered alone significantly increased the body weight gain as compared to normal control (Table 1).

### 3.2. Effects of *C. zeylanicum* Stem Bark Aqueous Extract on Hematological Parameters

Table 2 shows the effects of AECZ on some hematological parameters. Apart from the number of monocytes, no other hematological parameter varied significantly regardless of the treatment received. Gentamicin significantly decreased by 52.95% (*p* < 0.001) the number of monocytes compared to the normal control. Silymarin (50 mg/kg) and AECZ at the doses of 200 and 400 mg/kg significantly increased the number of monocytes by 52.50% (*p* < 0.05), 90% (*p* < 0.001), and 67.50% (*p* < 0.01), respectively, compared to the group treated only with gentamicin.

### 3.3. Effects of the Aqueous Extract of *C. zeylanicum* Stem Barks on Serum Glucose, Albumin, Total Protein, and Aspartate Aminotransferase

The effect of AECZ on serum glucose, total proteins, albumin, and aspartate aminotransferase (ASAT) is presented in Table 3. Consecutive to gentamicin administration, a significant decrease of proteinemia (25%) as compared to normal control was observed. Compared to GM group, AECZ at the doses of 200 mg/kg and 400 mg/kg significantly inhibited the decrease of proteinemia by 17.30% (*p* < 0.05) and 23.07% (*p* < 0.01), respectively. In the GM group, the serum concentration of albumin increased significantly by 15.91% (*p* < 0.001) compared to normal control group, but concomitant treatment with GM and AECZ at the doses of 200 and 400 mg/kg significantly (*p* < 0.001) reduced the serum albumin by 12.83 and 13.73, respectively. The serum concentration of aspartate aminotransferase increased significantly by 57.7% (*p* < 0.001) in the GM group when compared to normal control. The administration of *C. zeylanicum* extract at the doses of 200 and 400 mg/kg or silymarin (50 mg/kg) resulted in a significant reduction in ASAT activity, respectively, by 52.71% (*p* < 0.01), 64.70% (*p* < 0.001), and 53.38% (*p* < 0.01) as compared to gentamicin-intoxicated rats. None of the evaluated parameters were affected by AECZ when administered alone.

### 3.4. Effects of the Aqueous Extract of *C. zeylanicum* Stem Barks on Some Kidney Biomarkers and Electrolytes

The effects of AECZ on kidney's function were evaluated by determining the rate of creatinine, uric acid, urea, and electrolytes (calcium, potassium, sodium, and chloride ions) in serum (Table 4). Gentamicin significantly increased serum creatinine by 77.72%, urea by 131.14%, and uric acid by 123.03% and decreased calcium level by 56.56% as compared to normal control. In comparison to negative control group, the two doses of AECZ (200 mg/kg and 400 mg/kg) significantly (*p* < 0.001) inhibited the increase of serum creatinine by 43.72% and 47.16%, urea by 57.08% and 47.16%, and uric acid by 57.08% and 47.16%, respectively. AECZ at the doses of 200 mg/kg and 400 mg/kg also prevented the decrease of calcium level by 122.48% and 116.06%, respectively. Likewise, silymarin also prevented this decrease. The serum levels of potassium and sodium were not significantly affected by any of the treatments as compared to normal control.

### 3.5. Effects of the Aqueous Extract of *C. zeylanicum* Stem Barks on Renal Oxidative Stress Parameters

**Figure 1:**
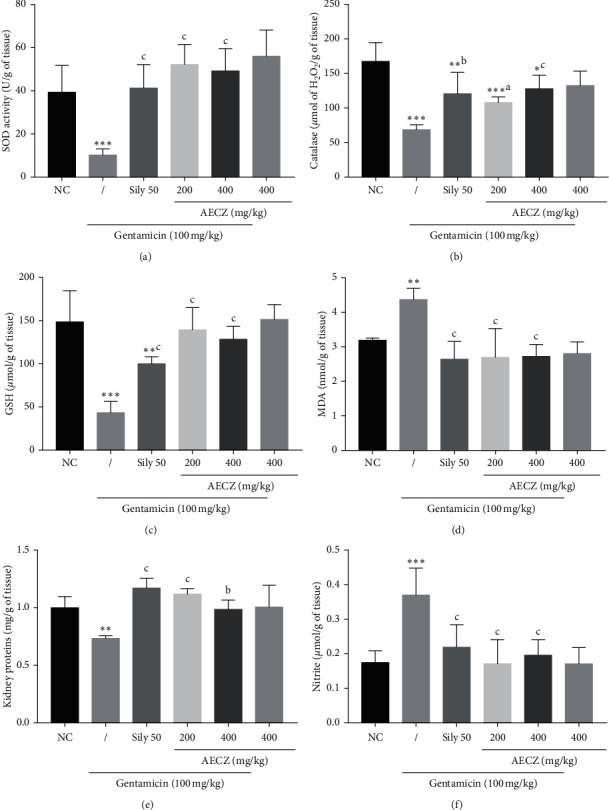
Effects of *Cinnamomum zeylanicum* stem bark aqueous extract on kidney's oxidative stress parameters. Each bar represents the mean ± SEM. *n* = 6; ^*∗*^*p* < 0.05, ^*∗∗*^*p* < 0.01, and ^*∗∗∗*^*p* < 0.001 indicate significant differences as compared to normal control. ^a^*p* < 0.05, ^b^*p* < 0.01, and ^c^*p* < 0.001 indicate significant differences as compared to the gentamicin group. NC: normal control, AECZ: stem bark aqueous extract of *Cinnamomum zeylanicum*, and Sily 50 = silymarin (50 mg/kg). (a) Superoxide dismutase. (b) Catalase. (c) Reduced glutathione. (d) Lipid peroxidation. (e) Proteins. (f) Nitrites.

The effects of the aqueous extract of *C. zeylanicum* stem barks on some kidney markers of oxidative stress are shown in Figure 1. The renal activity of SOD in the negative control decreased significantly (*p* < 0.001) by 73.79% (10.3 ± 1.14), compared with the normal control group (39.3 ± 5.05). However, pretreatment with silymarin (50 mg/kg) and AECZ (200 mg/kg and 400 mg/kg) significantly (*p* < 0.001) enhanced superoxide dismutase concentration by about 300.97%, 406.79%, and 377.66% (41.30 ± 4.45, 52.20 ± 3.78, and 49.20 ± 4.23, respectively) compared with the gentamicin group (10.30 ± 1.14).

Treatment with GM induced a significant decrease (*p* < 0.001) in the renal activity of CAT (68.60 ± 2.89) as compared to normal control rats (168.00 ± 11.00). Silymarin (50 mg/kg) significantly increased (*p* < 0.001) the activity of CAT in the kidneys by 76.38% as compared to the GM group (121.00 ± 12.7 versus 68.60 ± 2.89). AECZ at the doses of 200 mg/kg and 400 mg/kg significantly enhanced CAT concentration in a dose-dependent manner by about 57.43% (108.00 ± 3.33) and 86.58% (128.00 ± 7.91) compared to gentamicin group (68.60 ± 2.89).

An intraperitoneal injection of rats with gentamicin (100 mg/kg) for 14 days decreased the content of GSH significantly by about 70.87% (43.40 ± 5.43), compared to the normal control group (168.00 ± 11.01). Moreover, pretreatment with silymarin (50 mg/kg) or AECZ 200 mg/kg and 400 mg/kg one hour before gentamicin administration caused significant 2.3-, 3.2-, and 2.94-fold increases in renal glutathione content (100.00 ± 3.33, 139.00 ± 10.60, and 128.01 ± 7.91), respectively, compared with the gentamicin group (43.40 ± 5.43).

Gentamicin injection (100 mg/kg/day) for 14 days caused significant elevation (*p* < 0.01) of renal lipid peroxidation, which manifested as an increase in malondialdehyde concentration (4.37 ± 0.13), compared with the normal control group (3.19 ± 0.02). Pretreatment with silymarin (50 mg/kg) and AECZ (200 mg/kg and 400 mg/kg) for 14 days before gentamicin significantly reduced malondialdehyde concentration by about 38.58%, 38.21%, and 37.75% reaching 2.64 ± 0.21, 2.70 ± 0.33, and 2.72 ± 0.14, respectively, in comparison with the gentamicin group (4.37 ± 0.13).

Gentamicin-treated rats (negative control) showed significant decrease of 27% (*p* < 0.01) in kidney protein content as compared to normal control, whereas there was a significant increase in total protein content when animals were treated with silymarin (60.27%, *p* < 0.001), AECZ 200 mg/kg (53.42%, *p* < 0.001), and AECZ 400 mg/kg (35.61%, *p* < 0.01) as compared to GM-treated rats.

The level of nitrite was increased significantly by 112.64% (*p* < 0.001) in gentamicin-treated rats as compared to normal rats. This concentration of nitrite was decreased significantly by 40.81% (*p* < 0.01) in silymarin group, by 53.78% (*p* < 0.001) in AECZ (200 mg/kg) group, and by 47.02 (*p* < 0.001) in AECZ (400 mg/kg) group as compared to GM-treated rats.

### 3.6. Effect of the Aqueous Extract of *C. zeylanicum* Stem Bark on Proinflammatory Cytokines (TNF-*α*, IL-1*β*, and IL-6)

**Figure 2:**
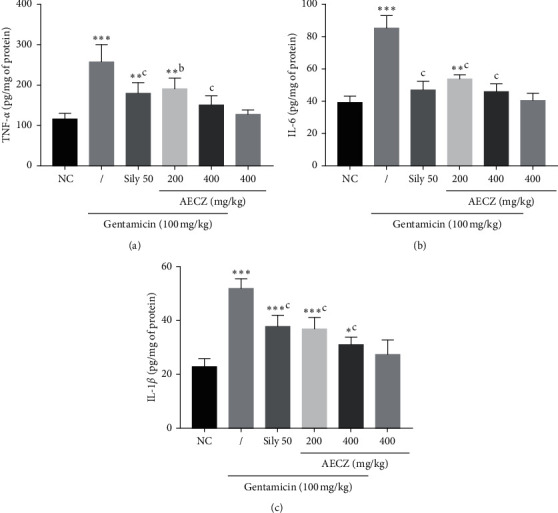
Effects of *Cinnamomum zeylanicum* stem bark aqueous extract on proinflammatory cytokines. Each bar represents the mean ± SEM. *n* = 6; ^*∗*^*p* < 0.05, ^*∗∗*^*p* < 0.01, and ^*∗∗∗*^*p* < 0.001 indicate significant differences as compared to normal control. ^b^*p* < 0.01 and ^c^*p* < 0.001 indicate significant differences as compared to the gentamicin group. NC: normal control, AECZ: stem barks aqueous extract of *Cinnamomum zeylanicum*, and Sily 50 = silymarin (50 mg/kg). (a) Tumor necrosis factor-alpha. (b) Interleukin-6. (c) Interleukin-1-beta.

As depicted in Figure 2, when compared to normal control group, GM administration induced a significant (*p* < 0.001) 2.21-fold elevation of TNF-*α* (116.0 ± 6.3 to 257.0 ± 18.9 pg/mg of protein). However, when these animals were concomitantly treated with silymarin (50 mg/kg) and GM, a 1.43-fold significant (*p* < 0.001) decrease in the TNF-*α* levels was noted. In groups treated with AECZ at the doses of 200 and 400 mg/kg, 1.34-fold and 1.70-fold decreases of the TNF-*α* levels were, respectively, observed.

GM administration induced a significant (*p* < 0.001) 2.17-fold elevation of IL-6 (from 39.3 ± 1.72 to 85.4 ± 3.45 pg/mg of protein) when compared to normal control rats. As compared to the GM-treated rats, silymarin (50 mg/kg) significantly (*p* < 0.001) decreased the IL-6 levels by 1.81 folds. In groups treated by AECZ at the doses of 200 and 400 mg/kg, significant (*p* < 0.001) 1.58-fold and 1.86-fold decreases of the IL-6 level were, respectively, observed.

Figure 2 also shows the effects of AECZ and silymarin on the levels of IL-1*β* in GM-treated rats. When compared to normal control rats, gentamicin induced a 2.26-fold significant (*p* < 0.001) elevation of IL-1*β* (from 22.9 ± 1.31 to 51.9 ± 1.6 pg/mg of protein).

As compared to GM group, silymarin (50 mg/kg) significantly (*p* < 0.001) decreased the IL-1*β* levels by 1.37 folds (from 51.9 ± 1.6 to 37.8 ± 1.85 pg/mg of protein). Treatment with AECZ at the doses of 200 and 400 mg/kg significantly decreased the level of IL-1*β*, respectively, by 1.40 folds (*p* < 0.01) and 1.66 folds (*p* < 0.001).

AECZ administered alone at the dose of 400 mg/kg did not significantly affect the cytokines' content, although a slight increase in IL-1*β* level was noted, when compared to the normal control.

### 3.7. Effects of *C. zeylanicum* Aqueous Extract on Renal Histology

**Figure 3:**
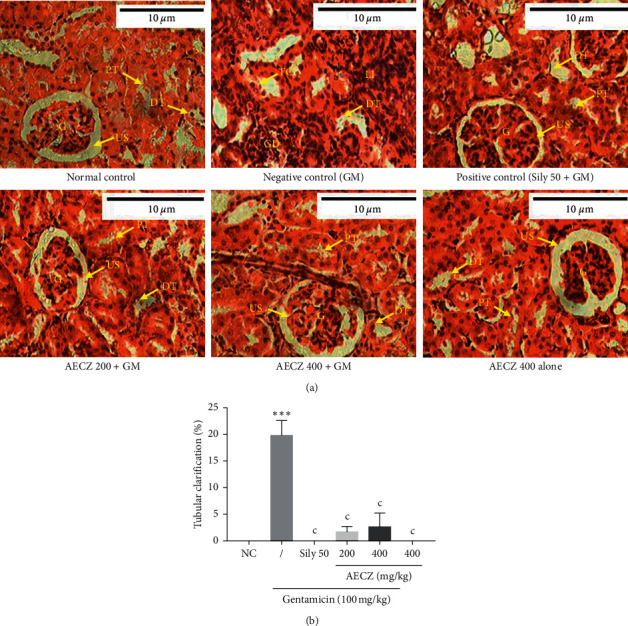
(a) Microphotographs of the kidney tissue (H&E, ×400) and (b) the percentage of tubular clarification. Each bar represents the mean ± SEM. *n* = 6; ^*∗∗∗*^*p* < 0.001 indicates significant difference as compared to normal control. ^c^*p* < 0.001 indicates significant difference as compared to the gentamicin group. NC: normal control, AECZ: stem barks aqueous extract of *Cinnamomum zeylanicum*, Sily 50 = silymarin (50 mg/kg), G: glomeruli, US: urinary space, PT: proximal tubule, DT: distal tubule, LI: leucocyte infiltrations, TC: tubular clarification, GD: glomerular degeneration, and GM: gentamicin.

Microscopically, kidney sections from control rats revealed the normal structure of renal parenchyma (Figure 3(a)). The kidney of rats in negative control group (Figure 3(a)) presented many structural alterations including leucocytes infiltration (inflammation), tubular necrosis, tubular clarification, cilia loss, glomerular degeneration, mesangial expansion, and significant reduction of urinary space. The architecture of the kidney of rats treated with AECZ at the doses of 200 mg/kg and 400 mg/kg as well as silymarin (Figure 3(a)) showed no histopathological alterations as compared to normal control group. Compared to the normal control group, gentamicin induced significant (*p* < 0.001) increase of tubular clarification (Figure 3(b)). The treatment with AECZ (200 and 400 mg/kg) as well as silymarin (50 mg/kg) reduced significantly (*p* < 0.001) this damage (Figure 3(b)).

## 4. Discussion

The purpose of this work was to evaluate the potential nephroprotective activity of the lyophilized aqueous extract of *Cinnamomum zeylanicum* stem bark in gentamicin-induced nephrotoxicity. AECZ and silymarin treatments prevented gentamicin's deleterious effects such as decrease in body weight, serum total proteins, and calcium level and increase in kidneys' relative weight, serum creatinine, urea, and uric acid. AECZ and silymarin also reduced oxidative stress status by increasing the levels of reduced glutathione, catalase, and superoxide dismutase activities and decreasing malondialdehyde, proinflammatory cytokines (TNF-*α*, IL-1*β*, and IL-6), and nitrites compared to gentamicin-treated group. Histological analysis of the kidney revealed that AECZ prevented kidney alterations induced by gentamicin.

Gentamicin has been intensively used as an experimental model of nephrotoxicity as it perfectly reflects clinical cases. The main aspect of gentamicin nephrotoxicity is tubular cytotoxicity. In fact, it causes tubular damage through necrosis of tubular epithelial cells predominantly in proximal segment and alteration of function of main cellular components involved in transport of water and solutes. In the present study, 14 days of gentamicin injection reduced body weight gain and increased the kidney's relative weight. The decrease of body weight gain could be a result of increased proteolysis and reduced proteins synthesis as previously demonstrated [35–37]. The proteolysis and reduced proteins synthesis were confirmed in this study through the decrease of the level of total proteins in the serum and kidneys of gentamicin-treated animal. AECZ significantly prevented the loss in body weight induced by gentamicin. Moreover, the animal treated with AECZ alone showed significantly higher weight compared to the normal control. It could then be suggested that AECZ prevents proteolysis or even potentiate protein synthesis. The hypothesis of inhibition of proteolysis is supported by the fact that AECZ significantly prevented the drop in serum protein content induced by gentamicin. However, the plant extract was unable to increase the serum protein concentration when administered alone, rolling out its probable effect on protein synthesis.

Oxidative stress activation and inflammatory process constitute a major part of gentamicin nephrotoxicity. In fact, nephrotoxicity induced by gentamicin is associated with the mitochondrial dysfunction in renal tubular cells. It causes a marked impairment in the activity of mitochondrial respiratory enzymes including NADH dehydrogenase resulting in the excessive ROS generation [38]. Furthermore, gentamicin has been reported to stimulate O_2_^–^ and NO^•^ generation through activation of inducible isoform of NO^•^ synthase (iNOS) in kidney [39] tissue. Reactive free radicals result in the lipid peroxidation, leading to the alteration of membrane lipid bilayer arrangement and reduction in antioxidant defense mechanisms, causing cellular damage and necrosis [39,  40]. AECZ significantly reduced the oxidative/nitrosative stress markers such as MDA and NO^•^ and further boosted the endogenous antioxidant parameters including SOD, catalase, and GSH, suggesting its *in vivo* antioxidant effects. This antioxidant effect of AECZ might contribute to its nephroprotective activity.

For more insight into the beneficial effects of AECZ, we evaluated additional parameters of nephron-cytotoxicity and renal function. Gentamicin induced significant increase in the blood levels of creatinine, urea, uric acid, albumin, and ASAT, indicating renal necrosis and dysfunction [1, 41]. Administration of AECZ or silymarin along with gentamicin caused significant decrease in these parameters. These results suggest that AECZ is able to protect the renal function and give additional evidence of nephroprotection.

The development of oxidative stress coupled with the necrosis triggers the inflammatory process [39, 40]. Inflammation was evidenced in gentamicin-treated animals by a drastic increase in proinflammatory cytokines (TNF-*α*, IL-1*β*, and IL-6).

The role of these proinflammatory mediators is well known in the kidney diseases [34,  42]. In this study, AECZ and silymarin significantly downregulate the renal proinflammatory cytokines, demonstrating the anti-inflammatory effect of this plant extract. These findings corroborate those of earlier studies, which proved that plant extracts are able to reverse the increase of proinflammatory cytokines levels induced by gentamicin [42–44]. Previous study on the hydroalcoholic extract of *Cinnamomum zeylanicum* stem bark on nociception and anxiety demonstrated that the anti-inflammatory effect is ascribed to *trans*-cinnamaldehyde component of cinnamon [25, 45].

Any toxic substance that triggers good functioning of the kidney provokes an inflammatory response in the kidney tissue, as well as its hypertrophy [46, 47]; the increase of the kidney's relative weight observed in animal group that received gentamicin could have probably resulted from the edema that was caused by drug-induced acute tubular necrosis. The plant extract as well as silymarin significantly mitigated the kidney hypertrophy. This may result from the anti-inflammatory potential of AECZ.

The haematopoietic system is one of the most sensitive targets for toxic compounds and an important index of physiological and pathological status in man and animal. Surprisingly, apart from the monocytes whose number decreased in animals treated with gentamicin, no other hematological parameter varied significantly independently of the treatment. This fact remains to be understood.

Aminoglycosides cause disturbance of renal functions by accumulating on the membranes of the proximal convoluted tubule, impairing the reabsorption and secretion of electrolytes and in that way causing homeostatic imbalance. In the present study, serum calcium level was reduced significantly and a nonsignificant decrease in serum sodium and potassium levels in gentamicin-treated rats was observed. These observations on serum sodium and potassium levels, although nonsignificant, are in agreement with those of Nafiu et al. [48] showing that serum sodium and potassium reduction was observed in gentamicin-treated rats, indicative of lesions in renal tubular epithelium and despair of Na⁺/K⁺-ATPase by gentamicin. Studies in animal and human have demonstrated that gentamicin and other aminoglycosides impair calcium transport in the renal tubules and, in some instances, result in a significant increase in urinary calcium excretion and in that way cause hypocalcemia [36]. AECZ as well as silymarin inhibited the decrease of serum calcium level in this study.

Evidences have indicated that the renal toxicity of gentamicin is due to its selective accumulation in the renal proximal convoluted tubules, which subsequently leads to the loss of the tubule brush border integrity, severe degeneration, necrosis in epithelial cells of the proximal tubules, and infiltration of mononuclear cells in intertubular spaces [4, 49]. In this study, the histological analyses of kidney sections from gentamicin-treated animals (negative control) showed inflammations, fibrosis, tubular clarifications, glomerular hypertrophy, and degeneration with a reduced urinary space. Previous studies described this renal tissue damage induced by gentamicin as well [36, 47]. The histological analysis of kidney sections from animals treated with AECZ or silymarin presented an aspect that is similar to that of normal animals. Compared to the normal control group, gentamicin induced significant increase of tubular clarification. The treatment with AECZ (200 and 400 mg/kg) as well as silymarin (50 mg/kg) reduced significantly this damage. This shows that AECZ prevented the damage of renal tissues by gentamicin. These histopathological observations correlate with the biochemical values. The animals that were treated with AECZ (400 mg/kg) alone showed no pathological changes. This indicates that AECZ does not possess any adverse effects. Interestingly, the two major constituents of *Cinnamomum zeylanicum* stem bark are cinnamaldehyde and eugenol. It has been well documented that they have various therapeutic properties like antioxidative and anti-inflammatory properties, as well as nephroprotective effects [25, 50–52]. Thus, the nephroprotective effect observed in the present study might be due to the presence of cinnamaldehyde and eugenol [45].

## 5. Conclusion

Data from the present study evidenced the nephroprotective effect of AECZ against gentamicin toxicity and further support the use of this plant by the local populations in the management of kidney diseases. The nephroprotective effect of AECZ is more likely mediated by its antioxidant and anti-inflammatory capacities. AECZ might be a good candidate for the development of new drugs that can protect against acute kidney diseases.

## Figures and Tables

**Table 1:** Effects of *C. zeylanicum* stem bark aqueous extract on body weight and kidney's relative weight.

	NC	GM	Sily 50 + GM	AECZ 200 + GM	AECZ 400 + GM	AECZ 400
BW D1 (g)	139.16 ± 8.93	143.33 ± 10.01	134.33 ± 8.81	139.00 ± 10.41	140.33 ± 5.91	140.66 ± 7.81
BW D14 (g)	179.88 ± 3.96	161.40 ± 6.78	174.61 ± 7.61	191.13 ± 8.79	178.50 ± 3.23	190.21 ± 4.13
ΔBW D14-D1 (%)	29.24 ± 1.49	15.81 ± 0.48^z^	33.03 ± 1.11^c^	39.05 ± 2.64^c^	33.66 ± 2.07^c^	40.51 ± 1.13^z^
Kidney's relative weight (g/100 g BW)	0.62 ± 0.01	0.92 ± 0.01^Z^	0.72 ± 0.01^c^	0.79 ± 0.01^c^	0.76 ± 0.02^c^	0.67 ± 0.01

Each value represents the mean ± SEM. *n* = 6; ^z^*p* < 0.001 indicates significant difference as compared to normal control. ^c^*p* < 0.001 indicates significant difference as compared to negative control. BW = body weight, ΔBW = variation of body weight, D = day, NC = normal control, GM = gentamicin, AECZ = stem barks aqueous extract of *Cinnamomum zeylanicum*, and Sily 50 = silymarin (50 mg/kg).

**Table 2:** Effects of *C. zeylanicum* stem bark aqueous extract on some hematological parameters.

Parameters	NC	GM	Sily 50 + GM	AECZ 200 + GM	AECZ 400 + GM	AECZ 400
WBC (×10^3^/*μ*L)	6.30 ± 0.67	5.26 ± 0.71	6.01 ± 0.94	7.25 ± 0.89	6.15 ± 0.87	6.20 ± 0.49
Lympho (×10^3^/*μ*L)	3.51 ± 0.22	2.68 ± 0.28	3.44 ± 0.51	4.00 ± 0.55	3.97 ± 0.49	3.47 ± 0.38
Mono (×10^3^/*μ*L)	0.85 ± 0.03	0.40 ± 0.04^z^	0.61 ± 0.03^a^	0.76 ± 0.06^c^	0.67 ± 0.06^b^	0.61 ± 0.05
Granulo (×10^3^/*μ*L)	1.45 ± 0.17	1.44 ± 0.11	1.15 ± 0.20	1.45 ± 0.20	1.24 ± 0.15	1.56 ± 0.08
RBC (×10^6^/*μ*L)	6.92 ± 0.14	6.77 ± 0.28	7.02 ± 0.10	7.09 ± 0.12	6.82 ± 0.18	6.88 ± 0.24
PLT (×10^3^/*μ*L)	472.6 ± 7.6	466.6 ± 46.6	458.1 ± 48.2	522.8 ± 48.2	457.5 ± 32.1	29.5 ± 35.7
Hb (g/dL)	13.31 ± 0.18	12.93 ± 0.64	13.25 ± 0.35	13.91 ± 0.20	12.53 ± 0.51	13.01 ± 0.54
HTC (%)	36.41 ± 1.41	34.41 ± 2.55	34.86 ± 1.85	36.50 ± 1.53	33.916 ± 1.09	35.65 ± 2.35
MCV (fL)	52.83 ± 2.56	50.70 ± 2.73	49.95 ± 2.48	53.60 ± 2.73	52.75 ± 2.24	51.97 ± 3.07
MCH (pg)	19.22 ± 0.39	19.02 ± 0.46	18.83 ± 0.40	19.95 ± 0.57	19.30 ± 0.28	18.88 ± 0.52
MCHC (g/dL)	36.73 ± 1.12	37.92 ± 1.14	37.98 ± 1.24	38.37 ± 1.21	36.92 ± 1.17	36.82 ± 1.30

Each value represents the mean ± SEM. *n* = 6; ^z^*p* < 0.001 indicates significant differences as compared to normal control; ^a^*p* < 0.05, ^b^*p* < 0.01, and ^c^*p* < 0.001 indicate significant differences as compared to the gentamicin group. NC: normal control, GM: gentamicin, AECZ: stem barks aqueous extract of *Cinnamomum zeylanicum*, Sily 50: silymarin (50 mg/kg), WBC: white blood cells, Lympho: lymphocytes, Mono: monocytes, Granulo: granulocytes, RBC : red blood cells, Hb: hemoglobin, PLT: platelets, HTC: hematocrit, MCH: mean corpuscular hemoglobin, MCV: mean corpuscular volume, and MCHC: mean corpuscular hemoglobin concentration.

**Table 3:** Effects of *C. zeylanicum* stem bark aqueous extract on serum glucose, total proteins, albumin, and aspartate aminotransferase.

Parameters	NC	GM	Sily 50 + GM	AECZ 200 + GM	AECZ 400 + GM	AECZ 400
Total proteins (mg/dL)	130 ± 0.02	104 ± 0.01^y^	120 ± 0.01^a^	122 ± 0.01^a^	128 ± 0.03^b^	129 ± 0.04
Albumin (mg/dL)	57.80 ± 1.05	67.00 ± 1.35^z^	57.50 ± 1.28^c^	58.40 ± 1.01^c^	57.80 ± 0.84^c^	56.50 ± 1.49
Glucose (mg/dL)	71.66 ± 7.04	68.04 ± 6.84	71.99 ± 6.37	73.60 ± 7.21	67.58 ± 3.63	65.73 ± 6.09
ASAT (UI)	172.54 ± 32.43	407.81 ± 79.83^z^	190.11 ± 27.84^b^	192.85 ± 23.56^b^	143.99 ± 20.18^c^	148.94 ± 7.08

Each value represents the mean ± SEM. *n* = 6; ^y^*p* < 0.01 and ^z^*p* < 0.001 indicate significant difference as compared to normal control. ^a^*p* < 0.05, ^b^*p* < 0.01, and ^c^*p* < 0.001 indicate significant differences as compared to negative control. NC = normal control, GM = gentamicin, AECZ = stem barks aqueous extract of *Cinnamomum zeylanicum*, Sily 50 = silymarin (50 mg/kg), and ASAT = aspartate aminotransferase.

**Table 4:** Effects of *C. zeylanicum* stem bark aqueous extract on some renal function biomarkers and electrolytes.

Parameters	NC	GM	Sily 50 + GM	AECZ 200 + GM	AECZ 400 + GM	AECZ 400
Creatinine (mg/dL)	0.57 ± 0.02	1.02 ± 0.09^z^	0.35 ± 0.05^c^	0.57 ± 0.04^c^	0.55 ± 0.03^c^	0.51 ± 0.05
Urea (mg/dL)	21.80 ± 1.73	50.39 ± 3.56^z^	20.89 ± 1.54^c^	26.49 ± 2.55^c^	21.69 ± 2.31^c^	25.13 ± 2.36
Uric acid (mg/dL)	2.44 ± 0.12	5.45 ± 0.87^z^	2.49 ± 0.12^c^	2.34 ± 0.15^c^	2.88 ± 0.14^c^	2.31 ± 0.14
Ca^2+^ (mg/dL)	4.82 ± 0.50	1.94 ± 0.15^y^	4.43 ± 0.37^a^	4.34 ± 0.67^a^	4.21 ± 0.78^a^	4.66 ± 0.54
K^+^ (mEq/L)	5.44 ± 0.25	4.70 ± 0.81	5.20 ± 0.36	5.52 ± 0.27	5.92 ± 0.49	5.50 ± 0.20
Na^+^ (mEq/L)	140.80 ± 1.15	134.00 ± 0.70	142.60 ± 0.67	135.20 ± 5.39	143.40 ± 0.50	141.00 ± 1.44
Cl^−^ (mEq/L)	105.80 ± 1.01	104.20 ± 1.20	104.00 ± 0.66	108.20 ± 0.96	105.20 ± 1.49	105.00 ± 1.14

Each value represents the mean ± SEM. *n* = 6; ^y^*p* < 0.01 and ^z^*p* < 0.001 indicate significant differences as compared to normal control. ^a^*p* < 0.01 and ^c^*p* < 0.001 indicate significant differences as compared to negative control. NC = normal control, GM = gentamicin, AECZ = stem barks aqueous extract of *Cinnamomum zeylanicum*, and Sily 50 = silymarin (50 mg/kg).

## Data Availability

All data supporting our findings are adequately contained within the manuscript.

## Abbreviations

AECZ: *Cinnamomum zeylanicum* stem bark aqueous extract
ANOVA: One-way analysis of variance
ASAT: Aspartate aminotransferase
CAT: Catalase
GD: Glomerular degeneration
DT: Distal tubule
EDTA: Ethylenediaminetetraacetic acid
G: Glomeruli
GM: Gentamicin
GSH: Reduced glutathione
H&E: Hematoxylin and eosin
Hb: Hemoglobin
HCT: Hematocrit
IL: Interleukin
LI: Leucocyte infiltrations
MDA: Malondialdehyde
NF-*κ*B: Nuclear factor-kappa B
NO: Nitric oxide
PT: Proximal tubule
RBC: Red blood cell
ROS: Reactive oxygen species
SEM: Standard error of mean
Sily 50: Silymarin (50 mg/kg)
SOD: Superoxide dismutase
TC: Tubular clarification
TNF-*α*: Tumor necrosis factor-alpha
UK: United Kingdom
US: Urinary space
USA: United States of America
WBC: White blood cell.
